# ^1^H HR-MAS NMR Based Metabolic Profiling of Lung Cancer Cells with Induced and De-Induced Cisplatin Resistance to Reveal Metabolic Resistance Adaptations

**DOI:** 10.3390/molecules26226766

**Published:** 2021-11-09

**Authors:** Martina Vermathen, Hendrik von Tengg-Kobligk, Martin Nils Hungerbühler, Peter Vermathen, Nico Ruprecht

**Affiliations:** 1 Department of Chemistry, Biochemistry and Pharmaceutical Sciences, University of Bern, 3012 Bern, Switzerland; 2 Department of Diagnostic, Interventional and Pediatric Radiology, Bern University Hospital, University of Bern, 3010 Bern, Switzerland; Hendrik.vonTengg@insel.ch (H.v.T.-K.); Martin.Hungerbuehler@dbmr.unibe.ch (M.N.H.); 3 Department of BioMedical Research, University of Bern, 3008 Bern, Switzerland; 4 University Institute of Diagnostic and Interventional Neuroradiology, Bern University Hospital, University of Bern, 3010 Bern, Switzerland

**Keywords:** cisplatin, cisPt resistance, non-small cell lung cancer, NSCLC, HR-MAS NMR, metabolomics

## Abstract

Cisplatin (cisPt) is an important drug that is used against various cancers, including advanced lung cancer. However, drug resistance is still a major ongoing problem and its investigation is of paramount interest. Here, a high-resolution magic angle spinning (HR-MAS) NMR study is presented deciphering the metabolic profile of non-small cell lung cancer (NSCLC) cells and metabolic adaptations at different levels of induced cisPt-resistance, as well as in their de-induced counterparts (cells cultivated in absence of cisPt). In total, fifty-three metabolites were identified and quantified in the ^1^H-HR-MAS NMR cell spectra. Metabolic adaptations to cisPt-resistance were detected, which correlated with the degree of resistance. Importantly, de-induced cell lines demonstrated similar metabolic adaptations as the corresponding cisPt-resistant cell lines. Metabolites predominantly changed in cisPt resistant cells and their de-induced counterparts include glutathione and taurine. Characteristic metabolic patterns for cisPt resistance may become relevant as biomarkers in cancer medicine.

## 1. Introduction

While cisplatin (cisPt) is the main drug for treating non-small cell lung cancer (NSCLC) [1] that accounts for 84% of all lung cancer diagnoses [2], cisPt resistance poses a major clinical problem [3]. On the cellular level, several processes may contribute to cisPt resistance either alone, or concerted, including reduced cisPt uptake, increased cisPt excretion, intracellular deactivation of cisPt, or activation of DNA-damage repair and anti-apoptotic pathways [3,4]. However, the exact mechanisms accounting for metabolic adaptations in cisPt resistant cells are not well understood and therefore, to date the challenge of cisPt resistance has not been solved.

Inherent or acquired platinum resistance is a major obstacle to improving long-term outcomes in cancer therapy. Recently, several new resistance mechanisms have been described that can be divided into two groups [5,6,7]. The first group is characterized by inadequate uptake of platinum into cancer cells, resulting in a decreased amount of aquatized cisPt in the cytoplasm, which in turn decreases the amount of platinum-DNA adducts. The second group is characterized by the failure to induce apoptosis due to the formation of platinum-DNA adducts. Numerous approaches have been pursued to overcome cisPt resistance in cancer patients [5]. Unfortunately, none of these approaches could be implemented in the clinic so far [1].

Standardized in-vitro cell models with controllable quantitatively incremented cisPt resistance allow investigating metabolic processes associated with resistance mechanisms systematically. Recently, the stepwise generation of such cisPt resistant NSCLC-cells has been reported. Furthermore, after de-induction, i.e., removal of cisPt from the culture media for several months, a long-term stable resistance was retained [8]. The protocol to yield NSCLC-cells with increasing induced and de-induced cisPt resistance was applied in the current study for metabolic characterization.

Metabolomics is an efficient tool for the analysis of the physiological state of living systems providing a metabolic fingerprint based on the multivariate statistical combination of a vast number of known and unknown metabolites (variables). It is very sensitive to detect any perturbations of the physiological state caused, for example, by disease, drug interventions, or drug resistance developments. With nuclear magnetic resonance (NMR) spectroscopy, numerous small metabolites in complex samples like cells, tissue or foodstuff can be analyzed simultaneously and mostly non-invasively with high reproducibility. Both, structural identification and quantitation can be assessed. NMR-based metabolomics has, therefore, become a well-established method among others in the pharmaceutical and biomedical field [9,10]. While classical NMR relies on solutions, i.e., liquid samples or solutions of extracts, high-resolution magic angle spinning (HR-MAS) NMR spectroscopy allows to directly obtain well-resolved spectra from whole, intact biological samples like cells or tissue providing the basis for their metabolic characterization [11]. In particular, the impact of cisPt on the metabolic profile of various cancer cell lines has been addressed by HR-MAS NMR-based chemometric analyses and has led to the identification of biomarkers for treatment response [12,13,14]. Recently, the metabolic alterations in cisPt sensitive and resistant A2780 cells following treatment with Ruthenium complexes as alternatives to cisPt were shown [15,16].

In the current study, HR-MAS NMR metabolomics was applied to A240286S NSCLC-cells (A24), using the cisPt sensitive wild-type (wt) strain as controls and eight sublines with different levels of induced cisPt resistance, as well as their de-induced counterparts. The first aim was to identify metabolites in the HR-MAS NMR spectra. The second aim was to analyze the metabolic profiles with respect to potential markers of cisPt resistance, thus, providing complementary information on pathways and metabolic adaptations involved in resistance mechanisms. Finally, the question was addressed if the previously observed maintenance of cisPt resistance in de-induced NSCLC cells is also reflected in the metabolic profile.

To our knowledge, this is the first study systematically monitoring by HR-MAS NMR metabolomics the development of drug resistance starting from a parent cell line going step-by-step towards higher cisPt exposures and including a de-induction protocol.

## 2. Results and Discussion

**Figure 1:**
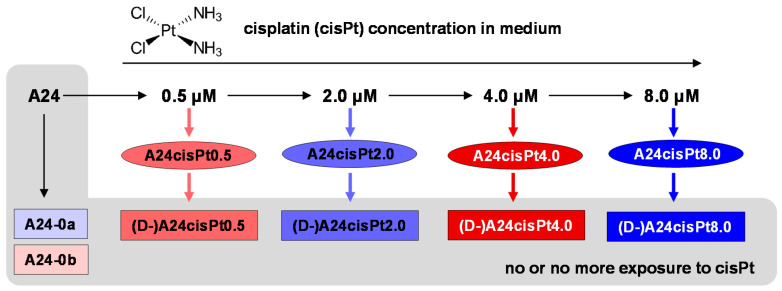
Overview of examined lung adenocarcinoma (A24) cell cultures prepared in two batches (red and blue) either not exposed to cisPt, exposed to increasing cisPt concentrations (ellipsoids) and de-induced (no more cisPt exposure for more than 3 months, rectangles). The color- and form-coding is maintained for all figures.

Figure 1 provides an overview of the A24 cell samples along with their sample labels used throughout this work that were included in the current metabolomic study. Overall, 30 cultured cell samples were prepared in two batches separated in time but applying an identical protocol (color-coded in red and blue, Figure 1). To induce cisPt resistance, the cells were exposed to increasing cisPt concentrations while for de-induction, cells were not exposed to cisPt anymore over more than 3 months [8]. For each cisPt concentration, resistant and de-induced cells were examined and labeled A24cisPt0.5 through A24cisPt8.0, and (D-)A24cisPt0.5 through (D-)A24cisPt8.0, indicated in Figure 1 with ellipsoids and rectangles, respectively; cisPt sensitive A24 wt cells not exposed to cisPt were used as controls (A24-0a, A24-0b).

### 2.1. The Metabolic Profile of A24 Cells

**Figure 2:**
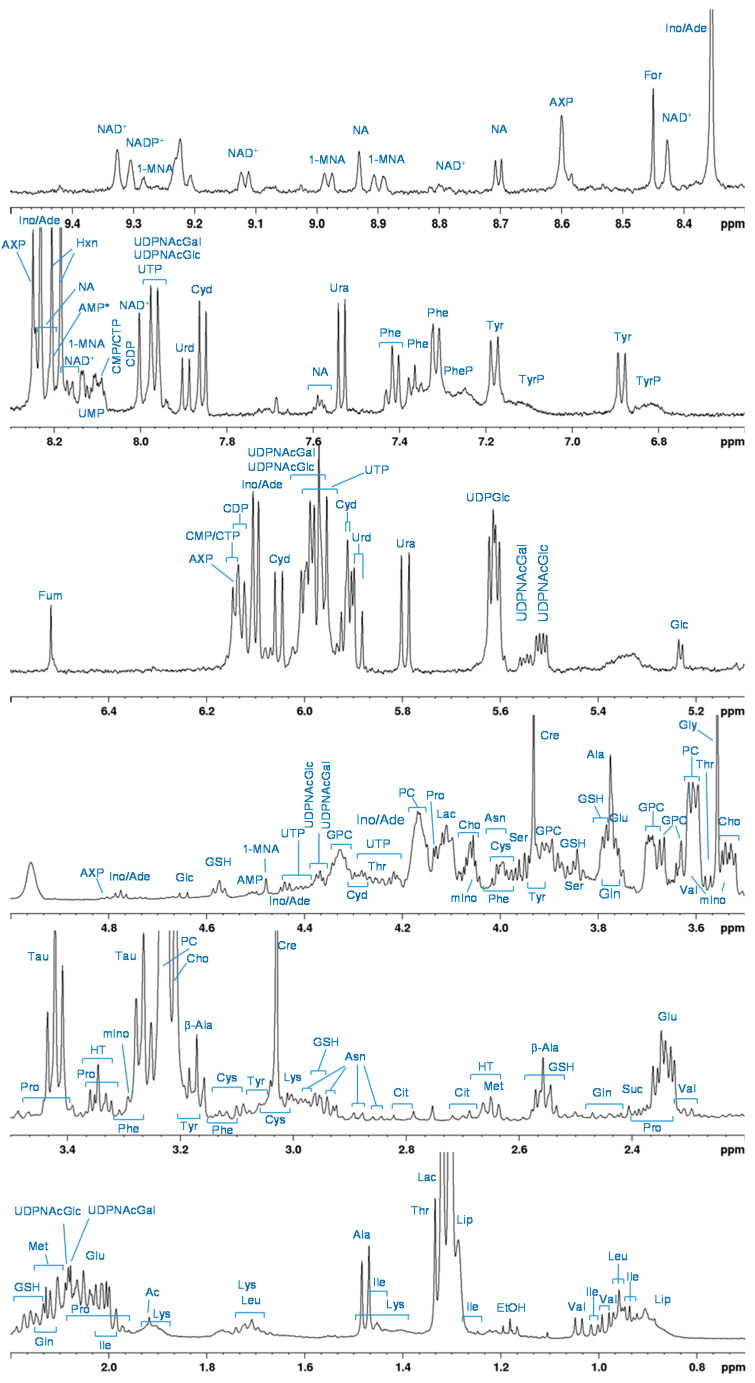
^1^H HR-MAS-PROJECT sum spectrum of A24 cell suspensions in PBS (T = 276 K) with resonance assignments. Spectral regions of 5–10 ppm were scaled up by a factor of 10.

Figure 2 depicts the ^1^H HR-MAS NMR 1D spectrum of A24 cells that were lysed and heated prior to acquisition to stop the metabolism and ensure stability during the experiment time. The T_2_-filtered spectrum mainly contains contributions from small metabolites whereas large molecules with short T_2_-relaxation times are suppressed. Overall, 53 different metabolites were identified in the ^1^H HR-MAS NMR A24 cell spectra. The assigned resonances are summarized in Table S1. The assigned metabolic profile of A24 cells consists of amino acids (total of 16 identified), small organic acids or salts thereof like acetate (Ac) and citrate (Cit), amines including creatine (Cre) and taurine (Tau), as well as several nucleobases, nucleosides, nucleotides and their corresponding phosphate sugars (UDPGlc, UDPNAcGlc/Gal). Further, resonances from glutathione (GSH), glucose (Glc), choline-containing compounds (Cho, PC, GPC) and lipids are visible. The appearance of lipid resonances that are not suppressed by the T_2_-filter most likely originate from mobile lipid pools, whereas in the non-T_2_-filtered TOCSY spectra (Figures S1–S4), a considerable portion of rather immobile lipids, including unsaturated lipids was also detectable (Figures S1 and S2). Resonance intensities in the downfield part of the spectrum (5–10 ppm) are about ten-fold lower than in the upfield part. Besides the resonances of the aromatic amino acids phenylalanine (Phe) and tyrosine (Tyr), broad resonances from their peptide homologs (PheP, TyrP) are also visible (Figure 2 and Figure S4) in the TOCSY spectrum. The unambiguous assignment of resonances to nucleotides and nucleosides, in particular to those derived from purine bases with two non-coupling singlets in the downfield spectral region, can be challenging. Their chemical shift values are in part difficult to distinguish (e.g., Ino/Ade) [17], are very pH-sensitive and often overlap for mono-, di- and triphosphates [18,19]. However, the resonance at 8.6 ppm labeled AXP most likely derives from AMP, since the adenine H-2-resonance of AMP separates quite well from that of ADP/ATP at neutral pH [18]. Most of the metabolites identified in the A24 cells have also been described in HR-MAS NMR spectra of lung cancer tissue [17,20]. In particular, high levels of Lac, PC, GPC and Cre are characteristic compared to normal lung tissue.

### 2.2. Principal Component Analysis (PCA)

**Figure 3:**
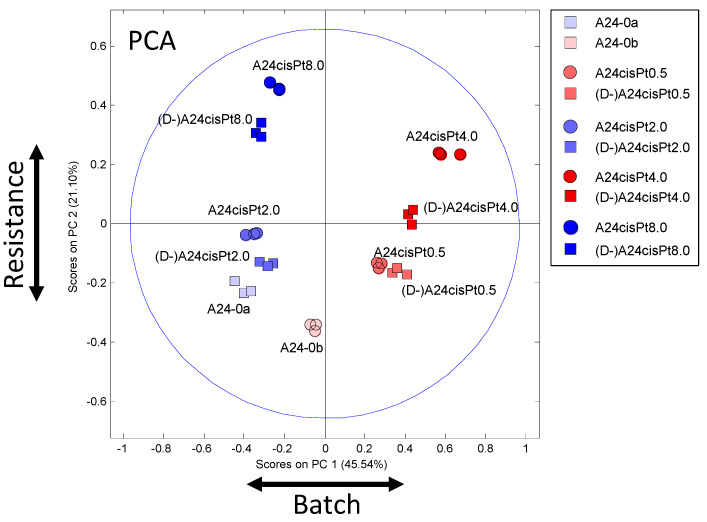
PCA scores plot (PC-1–PC-2) of all samples using the color and form coding introduced in Figure 1.

Principal component analysis (PCA) was applied to the whole data matrix (30 × 309) to probe in an unsupervised and unbiased way for potential clustering of the samples. The PCA scores plot explaining 45.5% and 21.1% of the total variance along PC-1 and PC-2, respectively, is shown in Figure 3. First, the PCA plot exhibits close clustering of replicates demonstrating high similarity and good reproducibility within sample groups. Second, samples with increasing resistance gain distance from the cisPt sensitive controls along PC-2. Third and most importantly, the de-induced samples are close to their induced counterparts demonstrating high metabolic similarity of cells exposed to the same cisPt concentrations. Finally, a clear separation of the two batches that were studied with a time lag between two experimental sessions (Figure 1) is visible along PC-1.

Despite the application of standardized procedures, batch effects [21] can be introduced that are often associated with variations inherent to biological material. They may originate from deviations of cell culture medium components, such as fetal bovine serum (FBS), or from minor changes in passage or harvest time. Nevertheless, the PCA plot demonstrates that NMR-based metabolomics is a very sensitive method for detecting subtle unintended distinctions among biological samples that translate into metabolic alterations. On the other hand, the method reproducibly reveals on top of that a metabolic response to external stimuli that is uncorrelated to the batch effect. Thus, the two effects could be clearly separated with the main direction of resistance along PC-2 and of batches primarily along PC-1.

### 2.3. Orthogonal Partial Least Squares Analysis (oPLS)

**Figure 4:**
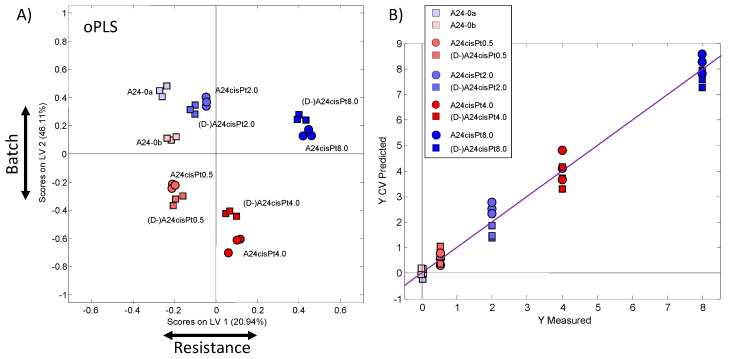
(**A**) oPLS scores plot (LV-1–LV-2) using the resistance as scaled prior knowledge (y-table). (**B**) Description of the PLS model showing the measured compared to the predicted resistance values.

To calculate a linear model for the prediction of cisPt resistance, orthogonal partial least squares (oPLS) analysis was applied to the data using the cisPt concentration in the culture media as dependent variables (Y-matrix). Similar results as in the unsupervised PCA (Figure 3) were obtained from oPLS analysis (Figure 4A): Close clustering of replicates and greater distance from the cisPt sensitive samples with increasing resistance were observed. The de-induced samples appeared close to their resistant counterparts indicating metabolic resemblance. Again, the two batches were clearly separated. Due to the oPLS guidance, the main direction of separation with resistance is switched compared to the PCA and is along LV-1, while the two batches are separated primarily along LV-2. Figure 4B demonstrates that the PLS model predicts the resistance of samples well: The predicted resistances are close to the measured ones. Robustness and predictive capability were very high with model quality parameters of R^2^ = 0.988 and Q^2^ = 0.982. Thus, the usage of different batches was of an advantage here, to demonstrate the robustness of the model that will also be applicable for potential future studies.

**Figure 5:**
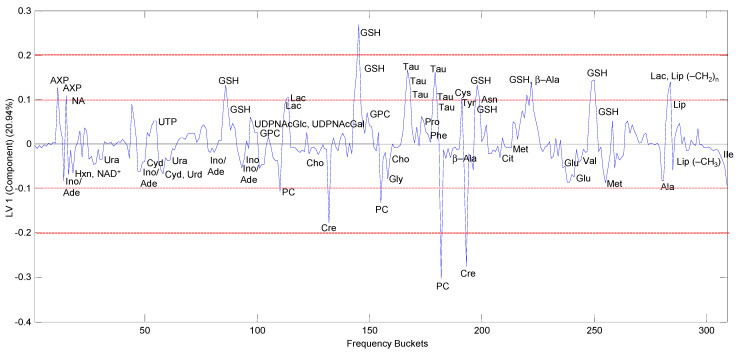
PLS-loadings of the first PLS component (LV-1), which was mainly separating the samples according to resistance. Positive LV components indicate higher metabolite concentrations in cells with increased cisPt resistance. The red lines are arbitrary lines marking thresholds of load values ±0.1 and ±0.2 above or below which metabolites are considered as strong contributors to separation.

The corresponding loading plot of the first PLS component (LV-1), which mainly separates the samples according to resistance, is shown in Figure 5. Positive LV components indicate higher metabolite concentrations in cells with increased cisPt resistance and vice versa. Metabolites with load values beyond an arbitrary threshold of ±0.1 and ±0.2 were considered to have a high or very high impact on class separation, respectively. Moreover, due to partial signal overlaps, the positive correlation of multiple buckets assigned to a specific metabolite was considered as a criterion for denoting a distinguishing feature. Accordingly, strong contributions marking cells with growing cisPt resistance could be attributed to increased concentrations of glutathione (GSH), taurine (Tau), adenosine-phosphate (AXP), and possibly lactate (Lac) that partly overlaps with lipids, while creatine (Cre) and phosphocholine (PC) appeared reduced.

The separation between batches was due to differences in Cho, lipid and especially PC levels. The corresponding loading plot of LV-2 is displayed in Figure S5. PC had a strong negative contribution on the second PLS component (LV-2) meaning that PC levels are elevated in batch b (red symbols in Figure 4). Because of this batch difference, Cho, lipid, and PC were excluded from further consideration of resistance features.

PCA and oPLS discriminant analysis (oPLS-DA) was additionally performed only on the samples with the same batch (Figure S6 shows this for batch a, similar results were obtained for batch b) and confirmed that GSH, Tau, AXP, and Lac are the main contributors to distinguishing samples based on resistance, whereas PC had little, or no impact on separation according to resistance (Figure S6 bottom).

### 2.4. Analysis of Single Metabolites

**Figure 6:**
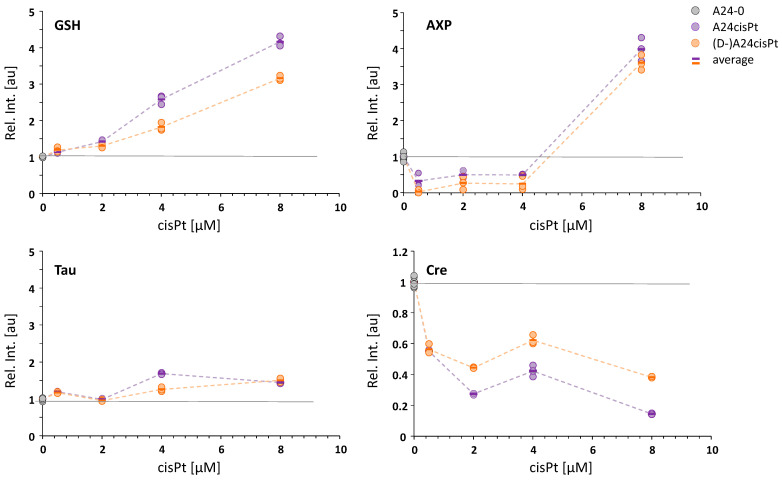
Metabolite levels relative to controls as function of cisPt concentration applied for resistance induction (purple: cells with induced resistance; orange: cells with resistance de-induced; gray: controls).

The metabolite levels mainly marking resistance are plotted relative to controls as a function of cisPt concentration for the samples with induced (purple symbols) and de-induced (orange symbols) cisPt resistance in Figure 6. Again, in these single metabolite graphs, it becomes evident that the metabolite concentrations are, throughout all samples, very similar in cells with induced and de-induced cisPt resistance. The most pronounced change was observed for GSH levels leading to a steady increase of up to a four-fold amount for the induced and three-fold amount for de-induced cells. AXP levels first decreased and significantly rose at cisPt concentrations of 8 μM. There was only a slight increase in lactate (Lac) levels, whereas the overlapping lipid methylene resonance (Lip (-CH_2_)_n_) did not contribute to the positive LV-1 loadings of Lac (Figure S7). Creatine (Cre) levels were strongly reduced down to 20% and 40% in induced and de-induced cells, respectively, compared to controls.

### 2.5. Metabolic Alterations Induced by cisPt Resistance

The deviation of the metabolic profile from cisPt sensitive cells increases with the concentration of cisPt exposure and most likely reflects the metabolic expression of increasing cisPt resistance previously proved by increasing IC_50_ values [8]. The metabolites Cre, GSH, and Tau that contribute with their altered levels to a cisPt resistance-specific profile were also found to be important for distinguishing lung adenoma-carcinoma from normal and other carcinoma tissue [20]. This indicates that these components not only play key roles specifically in the metabolism of lung cancer cells but also resistance development seems primarily expressed in the same processes where these marker compounds are involved.

#### 2.5.1. Glutathione (GSH)

Among the most striking metabolite changes was a significant increase in GSH levels in both induced and de-induced sublines with increasing cisPt resistance. Besides DNA-damage as the main cell death mechanism, cisPt also causes oxidative stress triggering the formation of reactive oxygen species (ROS) [22]. It is well known that the strong upregulation of GSH biosynthesis—due to enhanced activity of the Glu-Cys-ligase (γ-GCL)—is an important defense mechanism against oxidative insult through the drug where GSH functions as ROS scavenger [23]. In addition, high GSH levels may promote drug elimination from the cell via the multidrug resistance proteins (MRP family) whose transporting ability is co-regulated by GSH [24,25]. Formation of Pt-GSH complexes (Pt(GS)_2_) catalyzed by the enzyme GSH-transferase (GST) precedes MPR-mediated removal and in addition, competes with DNA-binding, thus, reducing DNA-damage. Based on these processes, increased GSH biosynthesis has been related to cisPt resistance [22,24,25] and is in good agreement with the correlation between the GSH amount and the extent of cisPt resistance found here (Figure 6). The presently observed high GSH levels indicate that GSH is not depleted by cisPt in cells with chronic cisPt exposure. Moreover, GSH is still provided in excess in cells a long time after suspension of chronic exposure reflecting cellular adaptation and metabolic reprogramming for maintaining the intracellular redox-balance [26]. In agreement with the current findings, significantly elevated GSH levels were recently reported in cisPt resistant ovarian A2780 cancer cells compared to the non-resistant parental cell line. The authors suggested a shift in glutamine (Gln) metabolism as the main source of resistance development since Gln synthetase activity—catalyzing Glu conversion to Gln—was suppressed favoring a redirection of Glu towards GSH synthesis [27]. However, a previous HR-MAS based metabolomic study of A2780 cells did not reveal significantly changed GSH levels as a distinct feature of cisPt resistance, which may be due to the fact that in this study, whole living cells were investigated where compartmentalized or bound GSH has reduced NMR visibility [15]. GSH increases reported in cisPt resistant versus sensitive head and neck squamous cell carcinoma (HNSCC) cells were related to metabolic reprogramming at a gene expression level. Several other metabolic pathways besides GSH synthesis were shifted and proposed to be involved in the resistance mechanism [28]. A recent transcriptomics study on the cisPt resistant NSCLC cell line A549 revealed the overexpression of four enzymes involved in the regulation of GSH and associated with resistance [29]. For cisPt *sensitive* cells, previous NMR metabolomics studies reported unchanged GSH levels in A549 lung cells [12], MG-63 osteosarcoma cells [13], and L02 human liver cells [30] in response to cisPt treatment. This was ascribed in part to the activation of cellular anti-oxidative reactions through GSH synthesis compensating GSH loss [12,13]. Unlike cisPt, ruthenium-based surrogates [15,16] and other anti-cancer drugs [31] induced increased GSH levels in cisPt-sensitive A2780 cells upon treatment. Since these drugs are proposed to follow alternative cell death pathways other than DNA-damage, their GSH consumption may be reduced.

In general, tumor cells often exhibit increased aerobic glycolysis that is reflected by changes in glucose and lactate levels [9,32] and known as the “Warburg effect” [33]. A downregulation of glycolysis with low dependence on Glc supply has been reported in lung cancer cells developing cisPt resistance [32,34,35]. This altered energy metabolism seemed specific for the type of cells since cisPt resistant ovarian cancer cells did not exhibit the same adaptations [32]. Similarly, Glc levels did not contribute to distinguishing cisPt sensitive from resistant cells and Lac levels were only slightly increased in the present study.

#### 2.5.2. Taurine (Tau)

Increased taurine (Tau) levels may reflect cellular defense and are reported to have anti-apoptotic effects in response to cisPt [36]. Tau plays a key role as a cellular osmolyte important for cell volume maintenance [37]. Its release is controlled by the volume-regulated anion channels (VRACs) of which the Leucine-rich repeat-containing protein 8 subunits LRRC8A and LRRC8D are specifically associated with Tau release [38,39]. The same protein subunits have also been suggested to be directly involved in the cellular uptake of cisPt via the VRACs and deficiency of these two proteins has been linked to the development of cisPt resistance [36,39]. Indeed, a clear downregulation of LRRC8D was previously observed in the cisPt resistant sublines used in the current study. However, LRRC8D was fully recovered in the corresponding—still cisPt resistant—de-induced sublines and LRRC8A was only reduced in A24cisPt-8.0 and (D-)A24cisPt-8.0 putting the role of these protein subunits for cisPt resistance into question [8]. Nevertheless, the lack of either LRRC8D or LRRC8A can impair Tau efflux leading to Tau accumulation in the cell [39], which may explain the 1.5-fold increase in Tau-levels for A24cisPt-4.0 (LRRC8D↓), A24cisPt-8.0 (LRRC8D/A↓) and (D-)A24cisPt-8.0 (LRRC8A↓) observed in the current study (Figure 6). Similarly, accumulation of Tau was found in cisPt resistant A2780 human ovarian cancer cells that correlated with resistance and reduced expression of LRRC8A [36].

**Figure 7:**
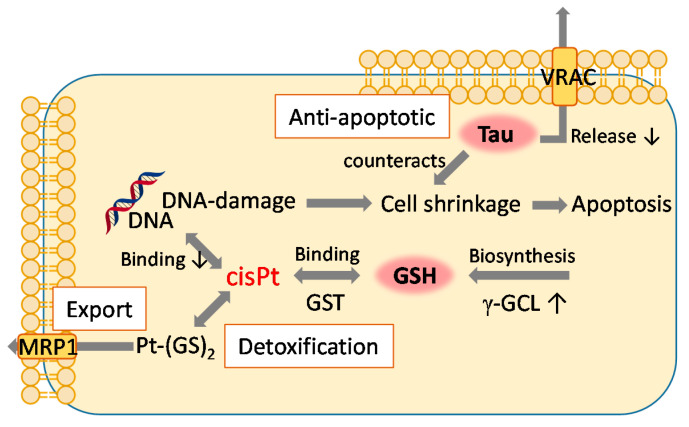
Proposed contributions from low molecular weight (MW) metabolites and processes involved in cisPt resistance. γ-GCL: Glu-Cys-ligase, GST: GSH-transferase, MRP1: multidrug resistance protein 1, VRAC: volume-regulated anion channel.

Figure 7 summarizes the potential routes of resistance mechanisms in which the two metabolites GSH and Tau with their elevated levels are proposed to be involved.

#### 2.5.3. Energy Balance Related Molecules

The initially reduced levels of AXP (most likely AMP, s.a.) strongly increased at exposures to the highest cisPt concentration (8 μM) in both induced and de-induced cell lines (Figure 6) marking a metabolic switch that reflects lasting adaptations in intracellular energy balance. A high ratio of AMP/ATP is a sign of energy shortage and activates pathways including the AMP protein kinase (AMPK) complex to maintain the cellular energy state. Moreover, adenylate kinase (AK) is an enzyme that catalyzes the conversion between ADP on one side and AMP and ATP on the other side and plays an important role in cellular energy metabolism. Overexpression of the isoform adenylate kinase 4 (AK4) has been associated with drug resistance including cisPt in cancer cells [40]. Located in the mitochondrial matrix, AK4 regulates mitochondrial activity and ATP supply and contributes to defense against oxidative stress. Upregulation of AK4 may thus explain elevated AMP levels that are concomitantly formed with ATP if the latter is directly consumed. In addition, reducing energy charge attenuates Cre influx through the Cre transporter in an AMP-activated protein kinase (AMPK)-dependent manner [41]. Cre is an essential component of cellular energy metabolism [42]. In both induced and de-induced sublines, a significant decrease in Cre levels was observed. The NMR visible sharp resonance at 3.03 ppm represents mobile, cytosolic Cre with a possible contribution of phosphocreatine (PCr). Even though Cre and PCr methyl NMR resonances can be resolved, PCr is often not observable due to fast hydrolysis [43]. The physiological conversion from PCr to Cre is catalyzed by the enzyme phosphocreatine-creatine kinase (CK) for efficient ATP supply [44]. The observed Cre and/or PCr reduction in both cisPt resistance induced and de-induced cells indicate sustained adaptations in the energy metabolism. The lower level of Cre in the cisPt resistant cells is in agreement with multidrug-resistant lung cancer cells [45] but in contrast to gemcitabine-resistant cells, in which the Cre level is elevated [46,47].

## 3. Materials and Methods

### 3.1. Chemicals

Cisplatin, *cis*-[PtCl_2_(NH_3_)_2_], (cisPt) was purchased in its pharmaceutical formulation (CISplatin Sandoz^®^, i.v. Infusion concentrate) from Galenica AG (Bern, Switzerland). Phosphate buffered saline (PBS, 50 mM, pH = 7.3) was prepared by mixing aliquots of 50 mM solutions of KH_2_PO_4_ and Na_2_HPO_4_ (provided by Sigma-Aldrich, Buchs, Switzerland) in H_2_O or D_2_O (99.9%, provided by Cambridge Isotopes Laboratories, Inc., Andover, MA, USA) containing 0.9 % NaCl.

### 3.2. Induction and De-Induction of cisPt Resistant Cell Lines

The cisPt sensitive wild-type (wt) A240286S cell strain (A24) and corresponding sublines with differently expressed cisPt resistance were cultured as previously described [8]. Briefly, A24 wt cells were exposed during several months to stepwise increasing cisPt concentrations in the culture medium. For this, aqueous solutions of cisPt were diluted with culture medium to yield final concentrations of 0.5 µM, 2.0 µM, 4.0 µM, and 8.0 µM cisPt (Figure 1). Beginning with the lowest concentration, each subsequent subline was obtained by splitting and propagating one-half of the cells for exposure to the next higher cisPt concentration. Newly established sublines were then exposed to their defining cisPt concentration for several months. For de-induction of cisPt resistance, cells from each subline were branched off and continued to be grown during several months in the absence of cisPt (Figure 1). CisPt sensitivity of all sublines was controlled by IC_50_ determination as previously described [8].

### 3.3. Preparation of Cell Samples for ^1^H HR-MAS NMR Spectroscopy

A24 cells and sublines were grown at 36.5 °C, 3.5% CO_2_ and humidified air in modified RPMI 1640 medium without riboflavin, phenol red and antibiotics, buffered with 4.5 mM HEPES (BioConcept, Allschwil, Switzerland), supplemented with 10% (*v*/*v*) fetal bovine serum (FBS) (Thermo Scientific, Waltham, MA, USA) and 13.5 mM NaHCO_3_. After approx. 24 h of growth after seeding, subconfluent cells were detached with Accutase (Thermo Scientific, Waltham, MA, USA), centrifugation (2000 rcf, 5 min), and removal of the medium followed by three washing steps with PBS (H_2_O). Finally, the cell pellet was suspended in 20 μL PBS (D_2_O), lyzed by shock freezing applying three alternating cycles of ultrasonication (30 s) and dipping into liquid nitrogen and stored at −70 °C until measurement.

All cell samples were prepared in two independent batches (a, b) at different time points (separated by 1 year) under the same conditions. Batch (a) included control samples (wt) and the sublines (induced/de-induced) at 2 μM and 8 μM cisPt levels; batch (b) included control samples (wt) and the sublines (induced/de-induced) at 0.5 μM and 4 μM cisPt levels (Figure 1). Each sample consisted of ~5x10^6^ cells and was prepared in triplicate resulting in a total of 30 (2 × 15) samples. Sample labeling was according to cisPt concentration and conditions applied as summarized in Table 1.

Prior to each measurement, cells were thawed and heated (20 min, 70 °C) to inactivate enzymes [48]. Cell suspensions were transferred into standard 4 mm ZrO_2_-MAS rotors using Teflon inserts for sample volumes of 50 μL and KelF rotor caps.

### 3.4. ^1^H HR-MAS NMR Spectroscopy–Data Acquisition

HR-MAS NMR experiments were performed on a Bruker Avance II spectrometer (Bruker BioSpin, Fällanden, Switzerland) operating at a ^1^H resonance frequency of 500.13 MHz. For magic angle spinning (MAS), a 4 mm HR-MAS dual inverse ^1^H/^13^C probe (Bruker BioSpin) with a magic angle gradient was used. The MAS rotors containing the cell samples were inclined by the magic angle (54.7°) and spun at a MAS rate of 3 kHz and a nominal temperature of 276 K. Temperature equilibration of the sample was ensured before acquisition. All samples within each batch were measured in random order and following a constant time protocol.

The TopSpin software (Bruker, version 3.2, patch level 5) was applied for data acquisition. ^1^H-NMR spectra were recorded using the 1D PROJECT (periodic refocusing of J evolution by coherence transfer [49]) pulse sequence with water pre-saturation and a T_2_-filter of 400 ms to suppress signals with short T_2_ (“*projectedpr1d*”). 512 scans were recorded over a spectral width of 6009.6 Hz (12 ppm) using 32 k data points, a relaxation delay of 4 s and an acquisition time of 2.73 s.

To support resonance assignments, 2D ^1^H^1^H TOCSY (total correlation spectroscopy) spectra applying the DIPSI2 pulse sequence (“*dipsi2phpr*” from the Bruker pulse program library) and 2-D ^1^H *J*-resolved experiments (“*jresgpprqf*” from the Bruker pulse program library) each with pre-saturation of the water resonance were acquired on a representative subset of samples.

### 3.5. Processing of ^1^H HR-MAS NMR Spectra

Spectral processing was performed using the Bruker TopSpin software (version 3.5b, patch level 7). To obtain the 1D PROJECT spectra, the FIDs were multiplied with an exponential window function using a line-broadening factor of 0.5 Hz before Fourier transformation. The spectra were phased and baseline corrected and chemical shifts were referenced to the -CH_3_ resonance of creatine (Cre) at δ = 3.03 ppm.

Resonance assignments were supported by 2D ^1^H^1^H-TOCSY and 2-D ^1^H *J*-resolved spectra, an in-house reference data bank, and further based on previously published data [15,16], as well as on the Human Metabolome Database 4.0 (HMDB) [50].

### 3.6. Data Analysis

The 1D PROJECT spectra were subdivided into 309 individually sized buckets within the spectral range of 0.8 ppm–9.5 ppm. The spectral region of 4.82 ppm–5.2 ppm containing the residual water resonance and noise regions were excluded. Buckets, i.e., integral regions, were manually selected to include isolated resonances where possible or otherwise to minimize overlaps. Integration on the single spectra was performed in TopSpin and coverage of all resonances was ensured by an overlay of a sum spectrum from the 30 single spectra. The integral regions of the 30 × 309 data matrix were exported for further statistical analyses as described below.

### 3.7. Multivariate Analysis of the 1D PROJECT Spectra

Multivariate analyses were performed using MATLAB R2019b (The MathWorks, Inc., Natick, MA, USA), PLS Toolbox (version 8.8.1, Eigenvector Research, Inc., Manson, WA, USA) and Excel (Microsoft Office 365 ProPlus, Redmond, WA, USA). To account for varying cell numbers, the bucket integrals were normalized by applying probabilistic quotient normalization (PQN). Further preprocessing of the data matrix included mean centering and pareto scaling. Unsupervised principal component analysis (PCA) and orthogonal partial least squares (oPLS) analysis using the SIMPLS algorithm [51] were performed. For the linear regression model using oPLS, the cisPt-concentration was applied as Y-variables. Cross-validation was performed applying Venetian blinds with five splits and one sample per split. In addition, the data were subjected to permutation testing. Model quality measures were calculated and measured-versus-predicted plots and loading plots were created in PLS-Toolbox.

### 3.8. Analysis of Single Metabolites

Integrals of buckets, either one or multiple, contributing to a specific metabolite, e.g., GSH, were averaged and used for single metabolite analysis. Only buckets with minimum overlap and high correlation were included as representative for single metabolites (GSH, Cre, and so on). The resulting averaged integrals for each sample class (A24cisPt0.5–(D-)A24cisPt8.0) were then referenced to the corresponding averaged integrals of the control group within each batch (A24-0a and A24-0b). Plots of relative metabolite levels as a function of cisPt concentration applied for resistance induction were created in Excel.

## 4. Conclusions

In the current study, eight independent NSCLC cell lines with different and stable levels of cisPt resistance and derived from the same parental cisPt sensitive cell line allowed a systematic approach addressing the development of cisPt resistance. The metabolic similarity of induced cisPt-resistant cells and their de-induced counterparts indicates an adjustment of the cells, along with a metabolic long-term memory. This is in agreement with the maintenance of cisPt-resistance reported in de-induced cells [8]. Accordingly, resistance is related to sustained molecular adaptations within the cells as was reflected in level changes of specific low MW components. Metabolites, such as GSH, Tau, and Cre may serve as biomarkers for cisPt resistance. The investigation of cell lines other than NSCLC cells with and without cisPt resistance will be useful in the future to extend and further validate the model and confirm the importance of the biomarkers elaborated in the present study.

The identification of marker compounds for cisPt resistance contributes to the knowledge of resistance mechanisms. This knowledge will be useful for the development of more effective anti-cancer drugs. While the metabolic profiling of cells rather provides a snapshot of the cell metabolome, additional studies analyzing the secretome would offer very useful complementary information on the flux of metabolites into and out of the cells. Moreover, detection of differences in the metabolism of cisPt resistant cells and their non-resistant counterparts may be of use for future studies of response to cisPt surrogates and other drugs. The potential resistance mechanisms indicated by the biomarkers, such as GSH synthesis, may serve as targets for modified drugs or for novel combinations of active ingredients to circumvent resistance.

## Figures and Tables

**Table 1:** Cell sample groups and their labels included in the study.

		cisPt Resistance	
Batch	Control	Induced	De-Induced
a	A24-0a	A24cisPt2.0	(D-)A24cisPt2.0
		A24cisPt8.0	(D-)A24cisPt8.0
b	A24-0b	A24cisPt0.5	(D-)A24cisPt0.5
		A24cisPt4.0	(D-)A24cisPt4.0

## Data Availability

The data presented in this study are available on request from the corresponding authors.

## Supplementary Materials

The following are available online. Figure S1: ^1^H^1^H-TOCSY (0.5 ppm–5.5 ppm) of A24 cell suspension in PBS with 1D PROJECT spectrum (A) and 1D NOESY spectrum (B) as projection in F2, Figure S2: ^1^H^1^H-TOCSY (0.5–2.5 ppm/0.8–5.4 ppm) of A24 cell suspension in PBS with assignment, Figure S3: ^1^H^1^H-TOCSY (2.4–4.8 ppm/0.7–5.0 ppm) of A24 cell suspension in PBS with assignment, Figure S4: ^1^H^1^H-TOCSY (5.4–9.5 ppm) of A24 cell suspension in PBS with assignment, Figure S5: PLS-loadings of the second PLS component (LV 2), which was mainly separating the samples according to batch. Positive LV components indicate higher metabolite concentrations in cells belonging to batch “a”, Figure S6: PCA and oPLS-DA with loading of the first PLS component (LV 1) only applied to the data of batch “a”, Figure S7: Metabolite levels of lactate (Lac) and lipid methylene (Lip (-CH_2_)_n_) relative to controls as function of cisPt concentration applied for resistance induction (purple: cells with induced resistance; orange: cells with resistance de-induced; gray: controls). Table S1: Resonance assignment of protons from A24 lysed cell suspension (PBS).

## Sample Availability

Samples of the compounds are not available from the authors.
