# Supplementary material for: 1H HR-MAS NMR Based Metabolic Profiling of Lung Cancer Cells with Induced and De-Induced Cisplatin Resistance to Reveal Metabolic Resistance Adaptations
Source: Molecules. 2021 Nov 9;26(22):6766. doi: 10.3390/molecules26226766 (PMC8625954; doi:10.3390/molecules26226766)
Supplement: Supplementary file 1 [file molecules-26-06766-s001.zip › molecules-1414162-supplementary.pdf]

Supporting Information

^{1}H HR-MAS NMR Based Metabolic Profiling of Lung Cancer Cells with Induced and De-induced Cisplatin Resistance to Reveal Metabolic Resistance Adaptations

Martina Vermathen [1,*,†], Hendrik von Tengg-Kobligk [2,3,†], Martin Nils Hungerbühler [2,3], Peter Vermathen [3,4,*,†] and Nico Ruprecht [2,3,*,†]

Content:

Table S1
Resonance assignment of protons from A24 lysed cell suspension (PBS).

Figure S1: ^{1}H^{1}H-TOCSY (0.5 ppm–5.5 ppm) of A24 cell suspension in PBS with (A) 1D PROJECT spectrum and (B) 1D NOESY spectrum as projection in F2.

Figure S2: ^{1}H^{1}H-TOCSY (0.5–2.5 ppm / 0.8–5.4 ppm) of A24 cell suspension in PBS with assignment.

Figure S3: ^{1}H^{1}H-TOCSY (2.4–4.8 ppm / 0.7–5.0 ppm) of A24 cell suspension in PBS with assignment.

Figure S4: ^{1}H^{1}H-TOCSY (5.4–9.5 ppm) of A24 cell suspension in PBS with assignment.

Figure S5: PLS-loadings of the second PLS component (LV 2), which was mainly separating the samples according to batch. Positive LV components indicate higher metabolite concentrations in cells belonging to batch “a”.

Figure S6: PCA and oPLS-DA with loading of the first PLS component (LV 1) only applied to the data of batch “a”.

Figure S7: Metabolite levels of lactate (Lac) and lipid methylene (Lip $(-CH_2)_n$) relative to controls as function of cisPt concentration applied for resistance induction (purple: cells with induced resistance; orange: cells with resistance de-induced; gray: controls).

Table S1. Resonance assignment of protons from A24 lysed cell suspension (PBS).

No	Compound	Abbr.	Chemical shift [ppm]	Group
Amino acids				
1	Alanine	Ala	1.477 (d)	β-CH_3
			3.788 (q)	α-CH
2	Asparagine	Asn	2.870 (m)	β-CH_2
			2.957 (m)	β-CH_2
			4.01 (m)	α-CH
3	Cysteine	Cys	3.065 (m)	β-CH_2
			4.007 (t)	α-CH
4	Glutamate	Glu	2.054 (m)	β-CH_2
			2.346 (m)	γ-CH_2
			3.769 (t)	α-CH
5	Glutamine	Gln	2.147 (m)	β-CH_2
			2.462 (m)	γ-CH_2
			3.78 (t)	α-CH
6	Glycine	Gly	3.557 (s)	α-CH_2
7	Isoleucine	Ile	0.937 (t)	δ-CH_3
			1.009 (d)	γ'-CH_3
			1.245 (m)	γ-CH_2
			1.454 (m)	γ-CH_2
			1.982 (m)	β-CH
			3.676 (d)	α-CH
8	Leucine	Leu	0.965 (d)	δ-CH_3, δ'-CH_3
			1.716 (m)	γ-CH, β-CH_2
			3.744 (t)	α-CH
9	Lysine	Lys	1.462 (m)	γ-CH_2
			1.708 (m)	δ-CH_2
			1.907 (m)	β-CH_2
			3.007 (t)	ε-CH_2
10	Methionine	Met	2.129 (s)	ε-CH_3
			2.167 (m)	β-CH_2
			2.641 (t)	γ-CH_2
			3.88 (t)	α-CH
11	Phenylalanine	Phe	7.317 (d)	2,6-CH
			7.367 (t)	4-CH
			7.411 (d)	3,5-CH
12	Proline	Pro	2.03 (m)	γ-CH_2
			2.355 (m)	β-CH_2
			3.339 (m)	δ'-CH_2
			3.413 (m)	δ''-CH_2
			4.14 (m)	α-CH
13	Serine	Ser	3.848 (m)	α-CH

			3.974 (m)	β-CH_2
14	Threonine	Thr	1.336 (d)	γ-CH_3
			3.591 (m)	β-CH
			4.262 (d)	α-CH
15	Tyrosine	Tyr	6.887 (d)	2,6-CH
			7.182 (d)	3,5-CH
16	Valine	Val	0.987 (d)	γ-CH_3
			1.041 (d)	γ’-CH_3
			2.257 (m)	β-CH
			3.616 (d)	α-CH
Peptides				
17	Glutathione	GSH	2.166 (m)	β-CH_2 (Glu)
			2.554 (m)	γ-CH_2 (Glu)
			2.958 (dd)	β’-CH_2 (Cys)
			3.780	α’’-CH (Gly)
			3.856 (t)	α-CH (Glu)
			4.577	α’-CH (Cys)
18	Phe-Peptide	PheP	7.25 (br)	2,6-CH
			7.371 (br)	3,5-CH
19	Tyr-Peptide	TyrP	6.817 (br)	2,6-CH
			7.107 (br)	3,5-CH
Organic acids				
20	Acetate	Ac	1.919 (s)	-CH_3
21	Citrate	Cit	2.649 (d)	-CH_2
			2.805 (d)	-CH_2
22	Formic acid	For	8.45 (s)	-CH
23	Fumarate	Fum	6.519 (s)	CH=CH
24	Lactate	Lac	1.32 (d)	β-CH_3
			4.125 (q)	α-CH
25	Succinate	Suc	2.405 (s)	-CH_2
Amines				
26	β-Alanine	β-Ala	2.558 (t)	α -CH_2 (-CO_2H)
			3.172 (t)	β-CH_2 (-NH_2)
27	Creatine	Cre	3.03 (s)	-CH_3
			3.934 (s)	-CH_2
28	Hypotaurine	HT	2.652 (t)	β-CH_2 (-NH_2)
			3.352 (t)	α -CH_2 (-SO_2H)
29	Taurine	Tau	3.265 (t)	-CH_2 (-SO_3H)
			3.422 (t)	-CH_2 (-NH_2)
Choline containing compounds				
30	Choline	Cho	3.209 (s)	-$N^+(CH_3)_3$
			3.534 (m)	N-CH_2
			4.076 (m)	O-CH_2
31	Glycerophosphocholine	GPC	3.234 (s)	-$N^+(CH_3)_3$
			3.634 (m)	-CH_2OH (glyc)

			3.677 (m)	-CH_2OH (glyc)
			3.699 (m)	N-CH_2
			3.901 (m)	-CHOH (glyc)
			4.343 (m)	O-CH_2
32	Phosphocholine	PC	3.225 (s)	-$N^+(CH_3)_3$
			3.613 (m)	N-CH_2
			4.18 (m)	O-CH_2
Nucleobases, nucleosides, nucleotides				
33	AXP (AMP)	AXP (AMP)	8.6 (s)	8-CH
			8.25 (s)	2-CH
			6.14 (d)	1’-CH (Rib)
			4.8 (t)	2’-CH (Rib)
			4.5 (m)	3’-CH (Rib)
34	CDP	CDP	7.97 (d)	6-CH
			6.12 (d)	5-CH
35	CMP/CTP	CMP/CTP	8.093 (d)	6-CH
			6.14 (d)	5-CH
36	Cytidine	Cyd	7.856 (d)	6-CH
			6.054 (d)	5-CH
			5.909 (d)	1’-CH (Rib)
37	Hypoxanthine	Hxn	8.184 (s)	8-CH
			8.205 (s)	2-CH
38	Inosine/Adenosine	Ino/Ade	8.231 (s)	8-CH
			8.355 (s)	2-CH
			6.10 (d)	1’-CH (Rib)
			4.776 (t)	2’-CH (Rib)
			4.438	3’-CH (Rib)
39	1-Methylnicotinamide	1-MNA	9.283 (s)	2-CH
			8.982 (d)	6-CH
			8.897 (d)	4-CH
			4.48 (s)	N^+-CH_3
40	NAD^+	NAD	9.328 (s)	2-CH (Nic)
			9.124 (d)	6-CH (Nic)
			8.804 (d)	4-CH (Nic)
			8.166 (t)	5-CH (Nic)
41	$NADP^+$	$NADP^+$	9.305 (s)	2-CH (Nic)
42	Nicotinamide	NA	8.933 (s)	2-CH
			8.704 (d)	6-CH
			8.235 (d)	4-CH
			7.582 (dd)	5-CH
43	UDP/UTP	UDP/UTP	7.968 (d)	6-CH (Ura)
			5.962 (d)	5-CH (Ura)
			5.984 (d)	1’-CH (Rib)
			4.375	2’,3’-CH (Rib)
44	UMP	UMP	8.119 (d)	6-CH (Ura)

			5.989 (d)	5-CH (Ura)
45	Uracil	Ura	7.534 (d)	6-CH
			5.797 (d)	5-CH
46	Uridine	Urd	7.897 (d)	6-CH (Ura)
			5.894 (d)	5-CH (Ura)
			5.922 (d)	1’-CH (Rib)
Nucleotide / phosphate sugars				
47	UDP-Glucose	UDPGlc	5.614 (m)	1-CH (Glc)
			4.083	2-CH (Glc)
48	UDP-N-Acetyl-galactosamine	UDPNAcGal	5.548 (dd)	1-CH (Gal)
			2.083 (s)	Ac-CH_3
			4.378	2’,3’-CH (Rib)
			5.972 (d)	5-CH (Ura)
			5.985 (d)	1’-CH (Rib)
			7.95 (d)	6-CH (Ura)
49	UDP-N-Acetyl-glucosamine	UDPNAcGlc	5.515 (dd)	1-CH (Glc)
			2.079 (s)	Ac-CH_3
			4.378	2’,3’-CH (Rib)
			5.972 (d)	5-CH (Ura)
			5.985 (d)	1’-CH (Rib)
			7.95 (d)	6-CH (Ura)
Lipids				
50	Lipid	Lip	0.908 (br)	ω-CH_3
			1.316 (br)	$(-CH_2)_n$
			1.347 (br)	$(-CH_2)_n$ unsat. lip.
			1.593 (br)	β-CH_2
			2.036 (br)	-CH_2-CH=
			2.293 (br)	α-CH_2
			2.836 (br)	CH=CH-**CH_2**-CH=
			4.30 (br)	-CH (glyc)
			5.338 (br)	-CH=CH-
Others				
51	Ethanol	Eth	1.182 (t)	-CH_3
52	Glucose	Glc	5.232 (d)	-CH_2
			4.648 (d)	
53	*myo*-Inositol	mIno	3.274 (t)	5-CH
			3.536 (dd)	1,3-CH
			3.616 (t)	4,6-CH
			4.056 (t)	2-CH

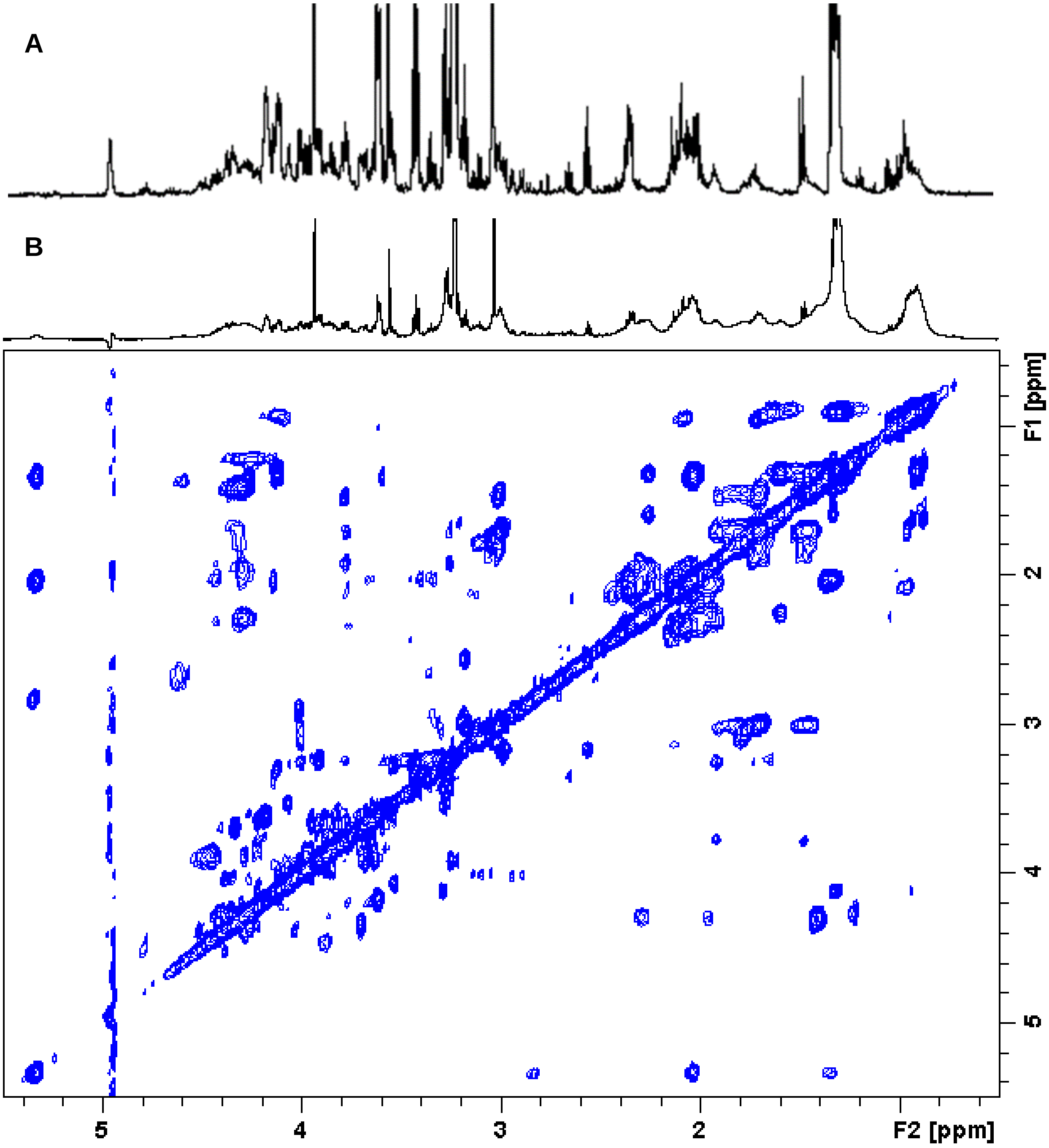

Figure S1: $^{1}H^{1}H$-TOCSY (0.5 ppm–5.5 ppm) of A24 cell suspension in PBS with (A) 1D PROJECT spectrum and (B) 1D NOESY spectrum as projection in F2.

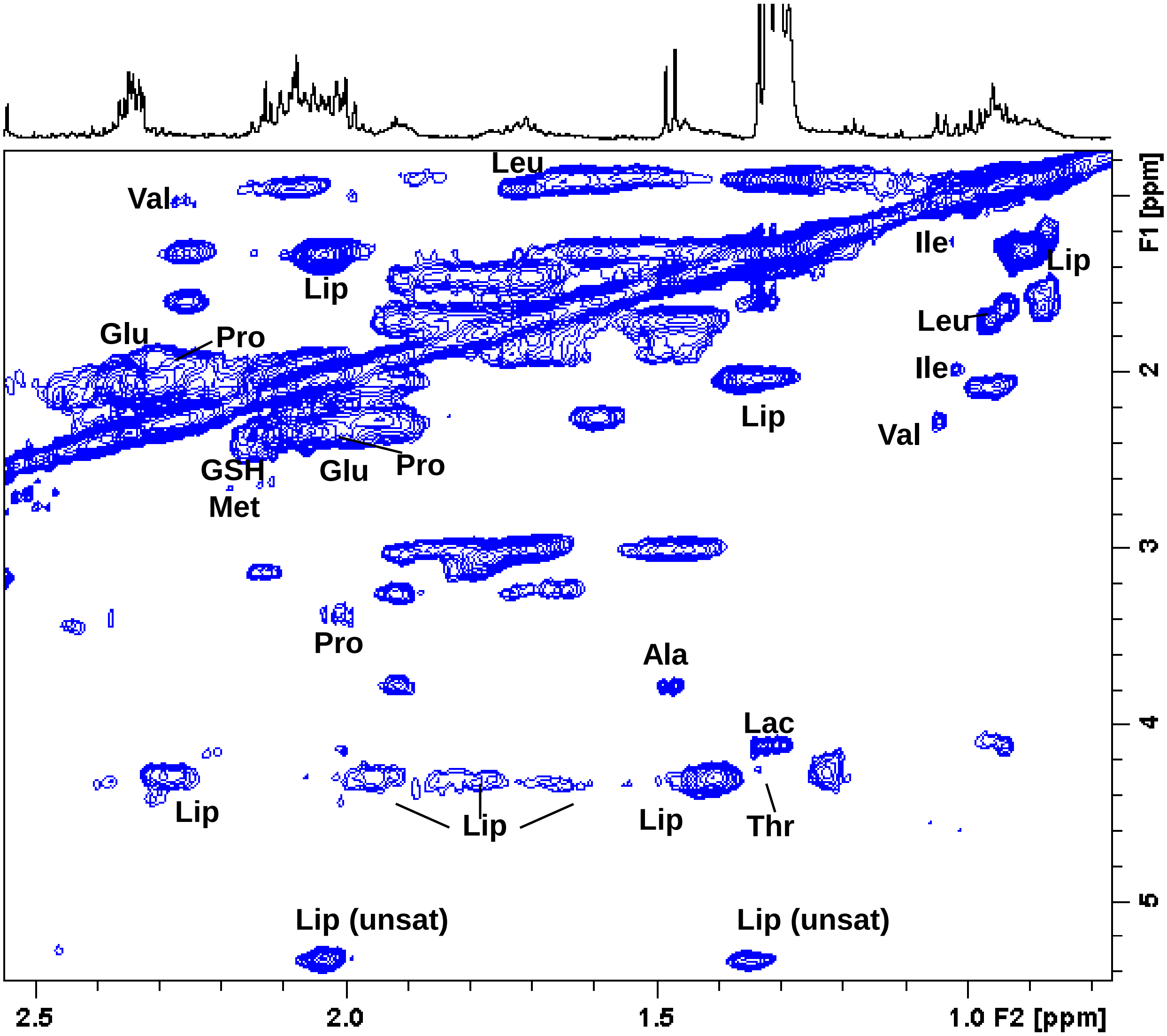

Figure S2: $^{1}H^{1}H$-TOCSY (0.5–2.5 ppm / 0.8–5.4 ppm) of A24 cell suspension in PBS with assignment.

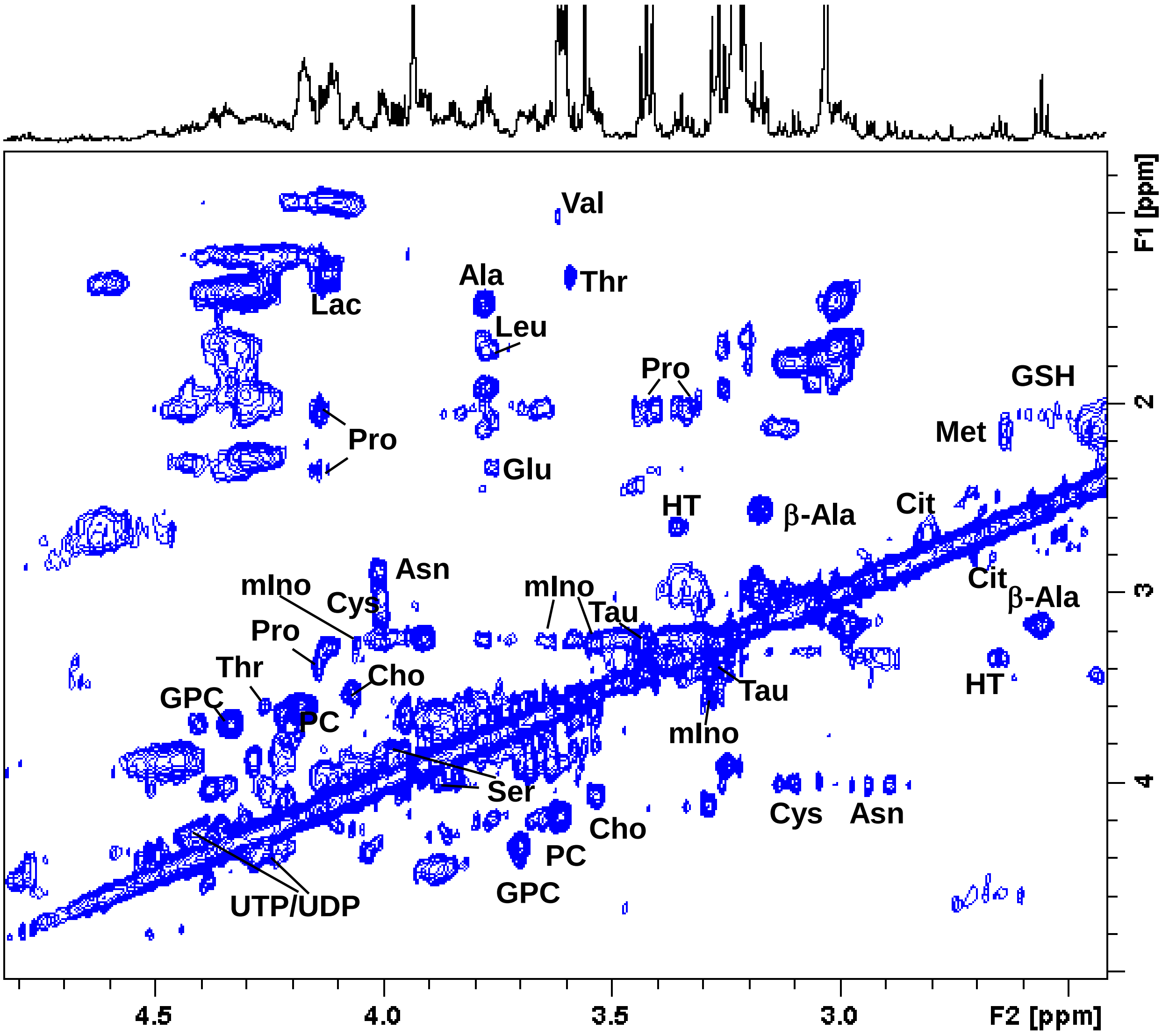

Figure S3: $^{1}H^{1}H$-TOCSY (2.4–4.8 ppm / 0.7–5.0 ppm) of A24 cell suspension in PBS with assignment.

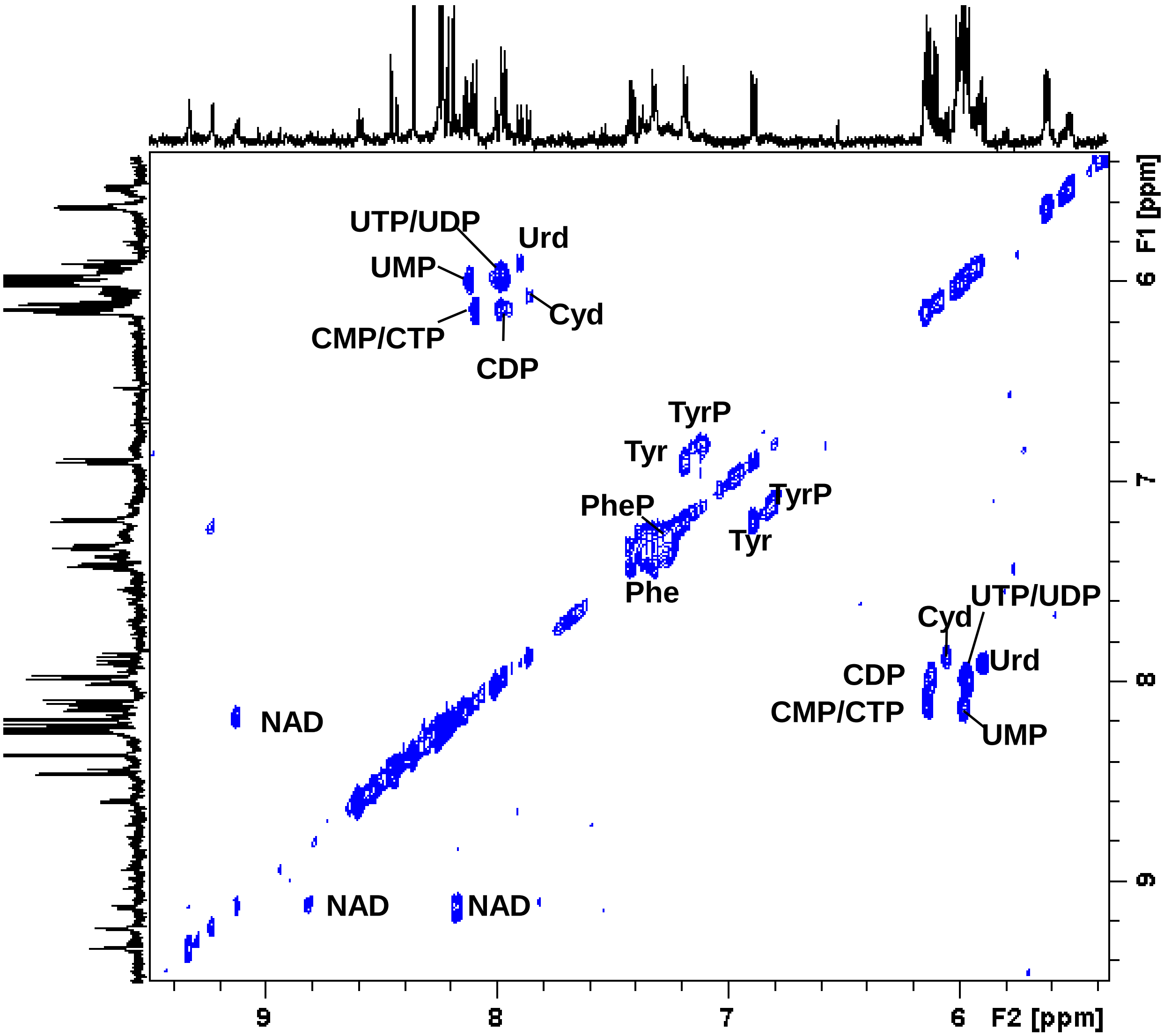

Figure S4: $^1H^1H$-TOCSY (5.4–9.5 ppm) of A24 cell suspension in PBS with assignment.

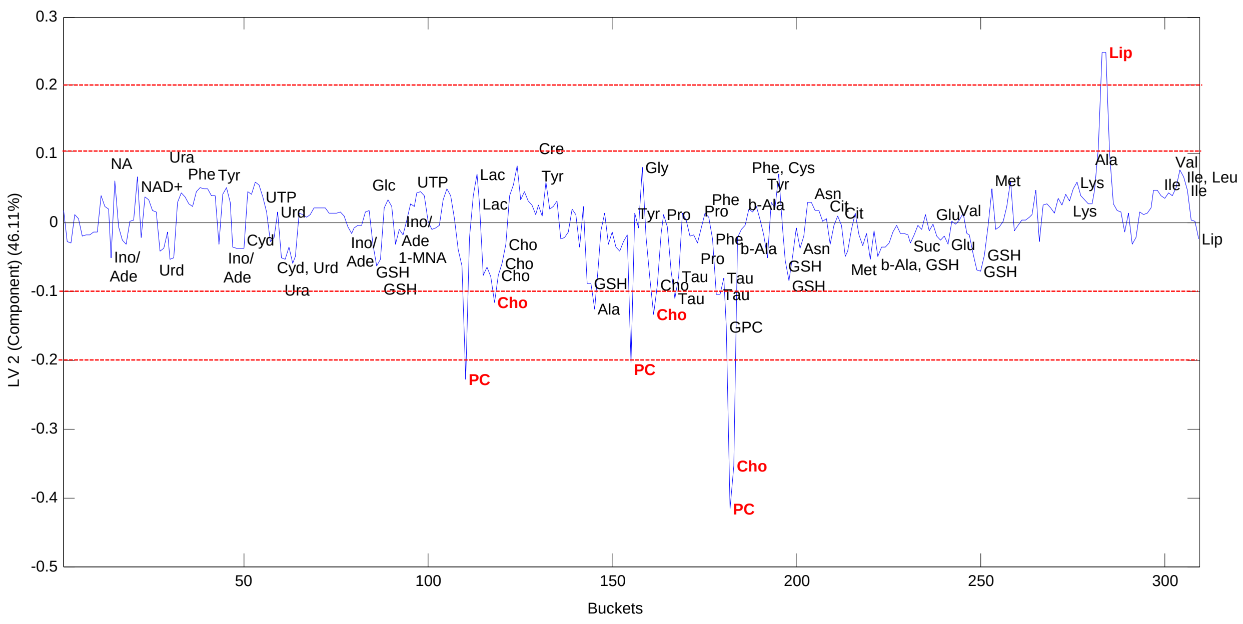

Figure S5: PLS-loadings of the second PLS component (LV 2), which was mainly separating the samples according to batch. Positive LV components indicate higher metabolite concentrations in cells belonging to batch “a”.

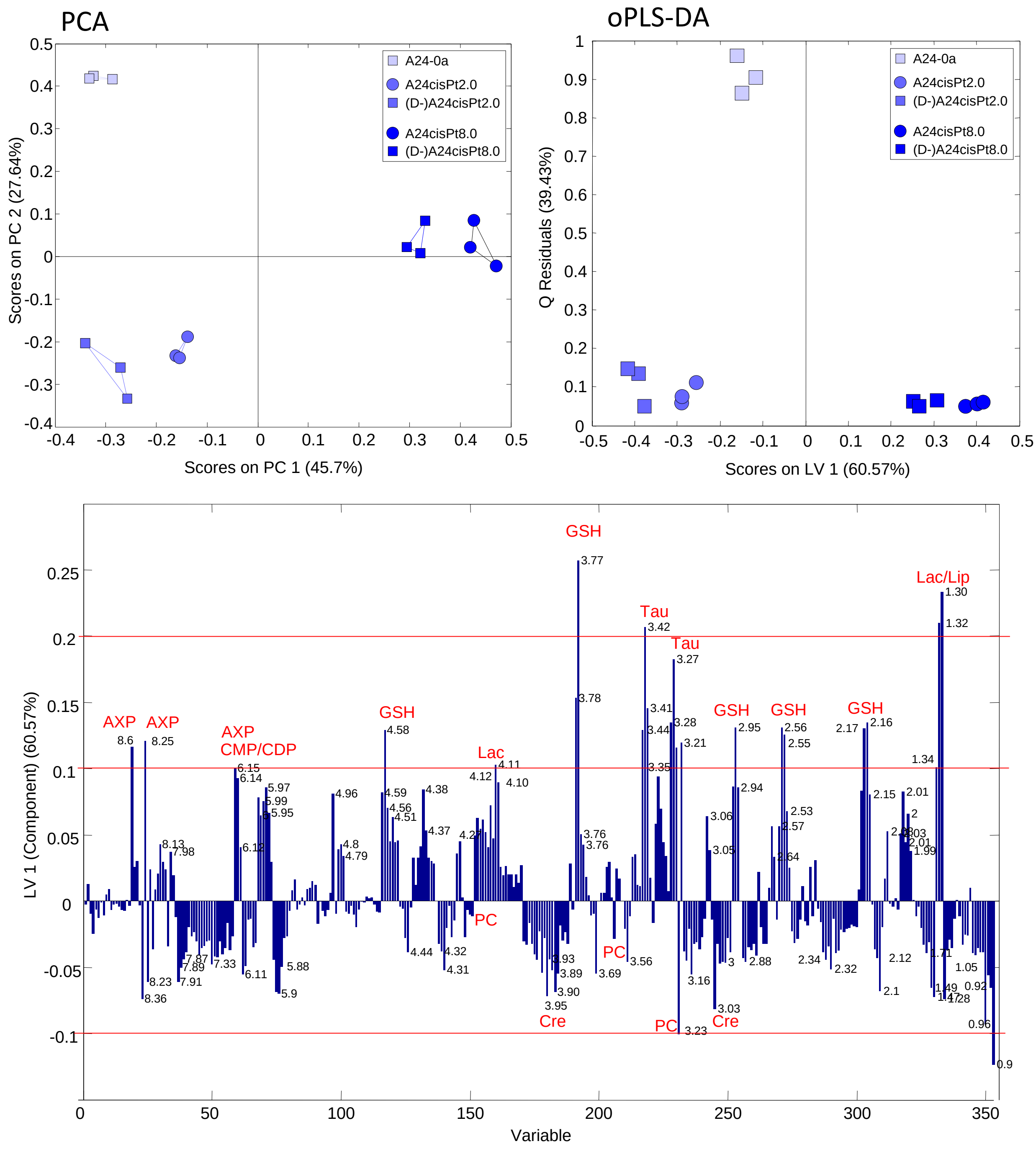

Figure S6: PCA and oPLS-DA with loading of the first PLS component (LV 1) only applied to the data of batch “a”.

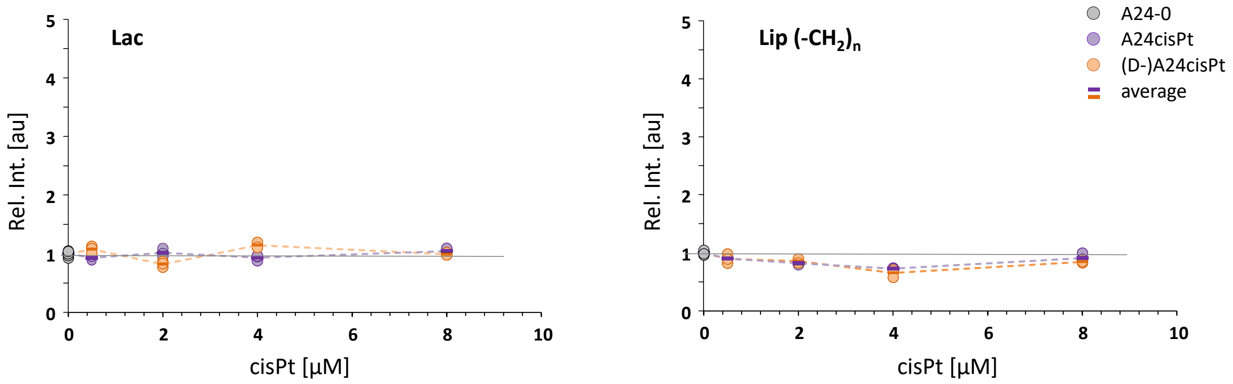

Figure S7: Metabolite levels of lactate (Lac) and lipid methylene (Lip $(-CH_2)_n$) relative to controls as function of cisPt concentration applied for resistance induction (purple: cells with induced resistance; orange: cells with resistance de-induced; gray: controls).
